# 

**Published:** 1911-05-20

**Authors:** 


### BOOKS RECEIVED.

John Wright and Sons, Ltd.

"The Medical Diseases of Children." By Reginald Miller, M.D.

Sanitary Publishing Company.

"Meat and Food Inspectors' Examinations." By Billing and Walker.

John Murray.

"Yellow Fever and its Prevention." By Sir Rubert Boyce, F.R.S.

Hodgetts, Ltd.

"Public Health Service Directory." By the Editor of Hodgetts.

Order of the Golden Age.

"The Cancer Scourge." By Robert Bell, M.D.

Surgery Publishing Company.

"Plaster of Paris, and How to Use It.' By M. W. Ware, M.D.

Royal Insurance Company, Ltd.

"Record of Sports."

It will be remembered that Sir John Barker offered £100 to the West London Hospital, if fifteen others would give £10 or thirty others £5 within a month. Half the required amount has been received and promised, but to enable the hospital to benefit by his offer £75 must be subscribed immediately.

The Duchess of Hamilton and Brandon will lay the commemoration stone of the Nurses' New Home of the London Homœopathic Hospital, Great Ormond Street, W.C., on Tuesday, May 23, at two o'clock, and open a sale of work at the Hospital at 2.30 o'clock. Two performances of Milton's Masque of "Comus" with the original music by Henry Lawes, will be given under the direction of Mr. Savage Cooper. Tickets of admission can be obtained of the Secretary, Mr. Edward A. Attwood, at the London Homœopathic Hospital, Great Ormond Street, W.C.

### BUILDINGS CONTEMPLATED AND IN PROGRESS.

BEAUMARIS.—For the building of a floating hospital at Beaumaris a tender of £1,068 has been accepted. The builders are Messrs. W. Thomas and Sons, of Amlwch, Port Anglesey. Mr. W. Owen, M.I.Mun.E., of Menai Bridge, is the architect.

BIGGLESWADE.—The Guardians of the Biggleswade Union require estimates for alterations and additions to the Infirmary at the Union Workhouse, to be delivered before June 6.

BRADFORD.—Architects submit designs before June 1 for the erection of a new infirmary in Duckworth Lane. Mr. Keith Young, F.R.I.B.A., is the assessor.

CROYDON.—A design by Mr. Frank Windsor, Licentiate R.I.B.A., M.S.A., submitted in competition has been accepted by the Committee for the King Edward Memorial Wing of the Croydon Hospital. It will cost about £4,000, of which £3,600 has already been subscribed.

CHARTHAM DOWNS, KENT.—For repairs, plastering, painting, etc., at the Asylum near Canterbury the Governors of the Kent County Lunatic Asylum seek tenders.

DUMFRIES.—Additions are contemplated to the Dumfries and Galloway Royal Infirmary at a cost of £11,660. Mr. G. R. Thomson, of Dumfries, is the architect.

GREENWICH.—Messrs. Young and Hall, F.R.I.B.A., are architects for the proposed extension of the Miller General Hospital, Greenwich Road. Many handsome donations have been made towards the cost, including £750 from the King Edward Fund. The extension will provide fifty-one additional beds, operating theatre, nurses' accommodation, etc.

HARTSHILL.—For the new out-patient department at the North Staffordshire Infirmary Mr. Keith D. Young, F.R.I.B.A., is architect. A tender of £9,482 by Mr. S. Wilton has been accepted. This was the lowest of eight tenders received, the highest amounting to £10,490.

LONDON.—Mr. A. A. Kekwick, architect, has received seventeen estimates for the additions and alterations at the receiving workhouse and casual wards, Lincoln's Inn Fields. The lowest amounts to £1,449, the highest to £1,890.

ROTHERHITHE.—The Bermondsey Guardians invite tenders for repairs and decorations at the Infirmary, Rotherhithe.

RYDE, ISLE OF WIGHT.—Mr. S. R. Cocks, of Ryde, architect, has just completed the isolation hospital there at a cost of about £6,700. Mr. John Meadow, of Cowes, was the contractor.

SHEFFIELD.—Tenders are invited for certain painting works at the Fir Vale Workhouse. Specifications may be had from the Clerk, Mr. A. E. Booker.

UPTON-ON-SEVERN.—Mr. Albert Baker, of Malvern, Worcestershire, is architect for the proposed extensions to the workhouse.

WALSALL.—For the erection of a bathroom, etc., at the hospital a tender of £252 has been accepted. Messrs. Bailey and McConnal are architects.

### JOTTINGS.

A STATE Cancer Hospital, to be managed by the State Commissioner of Health and six other trustees to be appointed by the Governor, is being established in Buffalo, U.S.A.

AT a joint meeting of the representatives of the Mountainside Hospital Association and the Montclair Homœopathic Society, an action was taken which will permit homœopathic physicians to practise in the wards of the hospital. Heretofore the homœopaths have had the use only of the operating room and the private rooms in the hospital, and plans have been considered for building a homœopathic hospital.

THE County of Surrey Memorial to King Edward has been so successful in raising funds that a clear £5,000—the sum originally aimed at—will be handed over at the end of May to the Children's Convalescent Home as an Endowment Fund.

PRINCESS HENRY OF BATTENBERG attended an entertainment at the Court Theatre last week in aid of the funds of the Queen's Hospital for Children. Its success must have been assured from the number of fashionable people and popular "stars," who took part or arranged the items. The programme consisted of a number of tableaux taken from popular plays, ancient and modern, which the audience recognised at once. There was also a children's ballet, and between the tableaux a variety concert, in which Lady Tree and Mr. George Grossmith, among others, took part.

### Gifts and Bequests.

The Bradford Royal Infirmary has received £200 from the executors of the late Mr. Jonathan Knowles.

Lord Mount Stephen has promised £5,000 to Dr. Gray's Hospital, Elgin, if Scotsmen in the county will provide a similar amount.

The sum of £1,008 has been received from the executors of the late Mrs. Christie for St. Mary's Hospital for Women and Children, Manchester, and Dr. D. Lloyd Roberts has received a promise of £1,000 for the endowment of a bed.

Mr. George Harding, of Liverpool, shipowner and merchant, who left estate of the gross value of £433,768, has left bequests to the following Liverpool institutions: £1,000 each to the Royal Infirmary, the Royal Southern Hospital, and the David Lewis Northern Hospital; £800 to the Seamen's Orphan Institution; £500 to the Stanley Hospital; and £100 to the Hospital for Women.

Mr. Henry Kirk, lace manufacturer, of Nottingham, has made the following bequests, free of legacy duty: £5,000 to the Nottingham General Hospital; £1,000 to the Nottingham General Dispensary; £300 each to the Nottingham Blind Institution, the Children's Hospital, the General Dispensary, and the Hospital for Women; £300 to the Mablethorpe Convalescent Home; £300 to the Skegness Convalescent Home; and £200 to the Nottingham Eye Infirmary.

### Medical Appointments, Etc.

At the monthly meeting of the Board, Dr. D. Dougal, M.B., Ch.B. Vict., was appointed resident surgical officer for the High Street Branch, Whitworth Park, of St. Mary's Hospital for Women and Children, Manchester.

At the meeting of the election committee of the Bradford Royal Infirmary held on Friday, Dr. Andrew Little was elected Honorary Laryngologist, vice Dr. Adolph Bronner, who was recently appointed Honorary Consulting Laryngologist.

### Appointments Vacant.

Full particulars of these vacancies can be obtained on reference to our advertisement columns:

Beckett Hospital, Barnsley.—Second House Surgeon.

Coventry and Warwickshire Hospital.—Junior House Surgeon.

Leicester Infirmary.—Second House Physician.

Macclesfield General Infirmary.—Senior House Surgeon.

Prince of Wales's General Hospital, Tottenham.—Honorary Physician and Junior House Physician.

Royal Albert Hospital, Devonport.—Assistant House Surgeon.

Wolverhampton and Staffordshire General Hospital.—House Surgeon.

### The Week's Novelties.

In order to commemorate the Coronation of their Majesties in June next, the Lord Mayor and citizens of Bristol intend presenting a box of chocolates to between seventy and eighty thousand school children. On the lid of the box are enamelled portraits of the King and Queen on a red ground, the background being of blue, the Royal Arms in gold on white, and the device of the rose, shamrock, and thistle suitably introduced. The execution of the order has been entrusted to MESSRS. J. S. FRY AND SONS, LTD., of Bristol and London, appointed manufacturers to their Majesties and to H.M. Queen Alexandra. This firm, whose chocolates have won a wide and well-deserved reputation for excellence and purity, has received large orders for similar boxes from other municipalities.

### Coming Events of the Week.

[Communications for this column must be sent to 29 Southampton Street, Strand, before noon on Wednesday.]

Saturday, May 20.—The Lord Mayor and Sheriffs visit the Lord Mayor Treloar's Cripples' Home at Alton, Hants.
Kelling Open-Air Sanatorium. Annual Meeting of Governors at the Guildhall, Norwich, 2.30 p.m.

Monday, May 22.—The Duchess of Norfolk lays the foundation-stone of the new hospital at Littlehampton.

Tuesday, May 23.—The Duke of Connaught opens the newly erected Research Institute at the Cancer Hospital, at 3 p.m.
Duchess of Norfolk opens a sale in aid of the University College Hospital, at 70 Ennismore Gardens, S.W.

Wednesday, May 24.—Annual General Meeting of the Asylum Workers' Association will be held at 11 Chandos Street, Cavendish Square, W., Sir William Collins in the chair, 2 p.m.

Thursday, May 25.—Middlesex Hospital, W. Court of Governors will be held to elect twelve Governors, 12 noon.
Princess Henry of Battenberg attends annual meeting of the Colonial Nursing Association, Lord Ampthill presiding, 3 Grosvenor Place, 3.30 p.m.

Friday, May 26.—London Homœopathic Hospital, Great Ormond Street. Lecture, "Latent Disturbances of Ocular Equilibrium, their Importance and Treatment," by C. Knox Shaw, 5 p.m.
West London Hospital, Hammersmith Road. Lecture, "Some Points in Treatment of Middle Ear Disease," by Dr. Davis, 5 p.m.

